# A One Health Approach to Public Safety: A Review of Police Canines in the United States

**DOI:** 10.3390/ijerph21091235

**Published:** 2024-09-18

**Authors:** Meera Gatlin

**Affiliations:** Department of Infectious Disease and Global Health, Cummings School of Veterinary Medicine, Tufts University, 200 Westboro Road, North Grafton, MA 01536, USA; meera.gatlin@tufts.edu

**Keywords:** public safety, animal-assisted intervention, police, law enforcement, canine, working dog, human–animal bond, public health, One Health

## Abstract

Working dogs are an essential part of modern society, and police canines (K9s) in the United States are amongst the most recognizable of all working dogs. Given the dearth of published data on active police canines in the U.S. and the calls for police reform, an interdisciplinary effort is necessary to comprehensively understand how these dogs are best used for the benefit of society. This review paper relies on veterinary public health expertise to present a comprehensive overview of police canine use by municipal law enforcement in the United States, including known impacts and gaps in knowledge. The existing literature from animal-assisted interventions (AAI) provides evidence to ensure working dog well-being, while human–animal bond research contributes to officer safety and canine partnership. Lastly, law enforcement and public health agendas (LEPH) assure the team’s successful efforts in built environments and local neighborhoods. These frameworks acknowledge the complexity of police dog use, spanning from punitive to public relations, which mirrors the role of law enforcement in U.S. society. This paper proposes the use of a One Health framework to ensure police canine contributions to society, including suggested approaches to partner municipal police with veterinary medicine and public health, and integrate One Health in promoting public safety for local communities.

## 1. Introduction to Police Canines

In the 10,000 years they have served as man’s best friend, dogs have taken on a variety of responsibilities alongside humans. In 2022, the American Veterinary Medical Association (AVMA) estimated that approximately 85 million dogs are owned in the United States, but these numbers come from surveys of pet owners [1]. Assuming these dogs are primarily kept for companionship, much less is known about working dogs in the United States. By definition, working dogs are tasked with a specific job, traditionally outside of companionship. Depending on the breed, responsibilities include hunting, livestock guardianship, herding, and transportation [2], but perhaps the most recognizable working dog in U.S. society is the police canine.

Police canines (stylized as K9s) or police dogs have been used in law enforcement for over 100 years, and traditionally, their responsibilities include scent detection, tracking people or objects of interest, and suspect apprehension [3]. Objects of interest include criminogenic commodities, such as explosives, firearms, and illicit drugs (i.e., narcotics), or may be articles associated with suspects, crime scenes, or forensic investigations. Apprehension or patrol canines are a non-lethal use of force [4] and are used to track and subdue suspects with physical apprehension (Technical terminology is adapted from: https://www.policeforum.org/assets/Canines.pdf, accessed on 31 May 2024); this may be conducted with contact (i.e., a bite), although this is not a necessity [5]. Other law enforcement canine roles and responsibilities have presently expanded to encompass arson (accelerant) detection, search and rescue, and even human remains recovery [2,6]. Perhaps the newest trend in police canines is the comfort dog (also called therapy or peer support dog), which provides emotional support and wellness to both officers and communities [7]. The most common breeds used in law enforcement are German Shepherd Dogs, Belgian Malinois, Dutch Shepherds, Labrador Retrievers, and German Short Haired Pointers. The herding breeds are popular for their high levels of motivation (“drive”) and agility skills, while the working breeds are selected for their olfactory capabilities, although a combination of these characteristics is noted in all the above [8].

The estimates on the number of working dogs in the United States vary, though numbers report as high as 50,000 [8]. The GAO reports approximately 5600 working dogs in the federal government, primarily employed in the Department of Homeland Security and Department of Defense [9]. While the U.S. military has published a significant amount of data on military working dogs (MWD) [10,11], law enforcement canines, particularly those employed by municipalities, differ in their capacity to protect and serve their local community.

Promoting and ensuring the beneficial use of animals in society are a major part of veterinary public health [12,13]. Police canines are unique in that their spectrum of work ranges from authoritarian and punitive to participatory and community-based [14]. On one end of the spectrum, police canines are used therapeutically (i.e., comfort or victim assistance), socially at community events, and to help find missing persons. Within these roles, police canines are valued and celebrated within local communities [15]. On the opposite end of the spectrum, police dogs are used for suspect apprehension, which can result in officer or suspect injury and may be less well-received by a community. This span of duties mirrors the role of law enforcement in society and an ideological shift to community policing. Rather than an assessment in isolation, a better understanding of how police canines are used in society requires integration with other healthcare professionals, reinforcing the concept of One Health. Given a larger movement to align law enforcement with public health agendas (known as LEPH), this review attempts to address the need for police canines using a One Health framework.

## 2. One Health to Safety

One Health is a collaborative and transdisciplinary approach that recognizes the intersection of health in people, animals, and our shared environment [16]. It recognizes that health is inextricably linked and that there is added value for the health of humans and animals, financial savings, environmental sustainability, and even social resilience, achievable by the cooperation of many disciplines [17]. Recent economic, environmental, and health crises have led to renewed recognition of this need for collaboration, positioning health professionals as agents of change [18]. One Health has been essential for addressing adverse health outcomes related to emerging zoonotic diseases and global pandemics, antimicrobial resistance, food security, and water safety, among others. However, it also lends itself to more than just biomedical concepts; fields like mental health, injury control and prevention, and even public safety can benefit from a One Health approach when appropriate disciplines and sectors collaborate [16].

Substituting health for safety when evaluating law enforcement is necessary, but exclusive determinants of safety are much less defined and often overlap with social determinants of health [19]. Public safety classically responds to crimes, disasters, and dangers and the threats of the above. If health is defined as the absence of disease or the promotion of healthy behaviors [20], then the analogy of safety implies an absence of crime or danger and the promotion of safe spaces. Safety should lead to a sense of physical, emotional, social, and material security, implying assurance and access with temporality and high probability.

**Figure 1:**
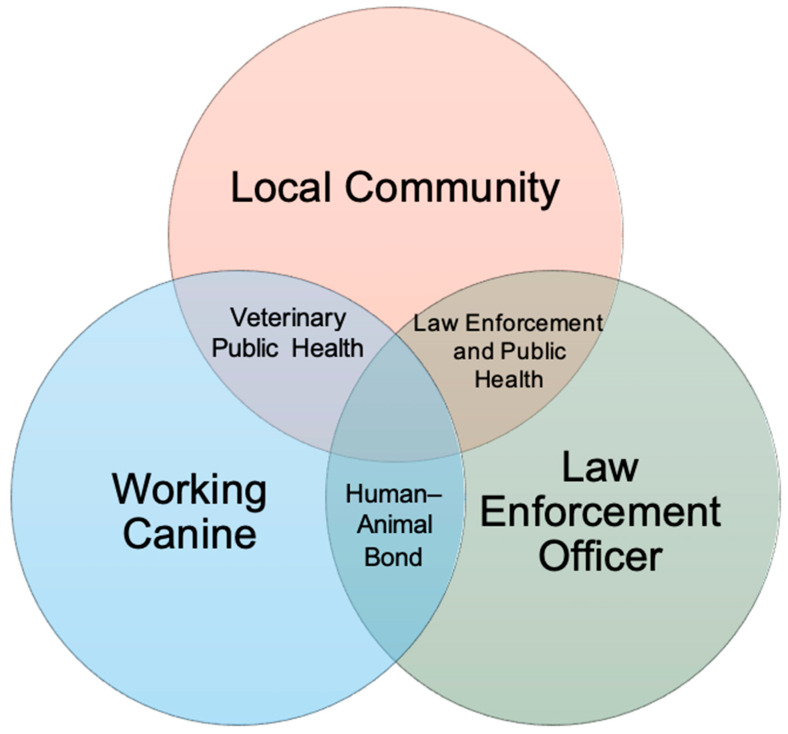
A One Health approach to public safety. Within circle intersections are suggested unifying frameworks.

It is important to address that, while public safety acknowledges the vital role that law enforcement plays, many have argued that police capacity is limited and that a deeper state of public safety and security can only emerge if upstream conditions, such as safe and affordable housing, are met [21,22]. The concerns around law enforcement violence have resulted in public health measures to promote police contact reduction and alternative response programs [23]. However, such upstream conditions cannot be readily addressed by this review, and this is recognized as a limitation. The inverse of this is the ability to address the institutionality of law enforcement by legitimizing their capability to protect and promote public safety and encouraging partnerships between police and public health. Applying One Health to police canines permits a focused reflection on working dog well-being, officer safety and canine partnership, and the duo’s efforts in built environments and local neighborhoods (Figure 1).

One Health transcends discipline boundaries and requires knowledge and perspectives from scientific and non-scientific sources to advance its mission [18]. If health and welfare challenges cannot be dealt with by a sole discipline, then this is arguably true for public safety as well. Therefore, essential partnerships are necessary for ensuring public safety using a One Health approach, for example, bringing together professionals for animal safety (veterinarians, particularly those with working dog expertise, and working dog trainers) and human safety (local law enforcement, clinical social workers, and police psychologists) and their shared physical environments (public health and public works). As with other disciplines, One Health practitioners organize multidisciplinary teams of experts from academia, government, and public institutions to generate awareness, policies, and practices to support and generate meaningful change for the benefit of society [18].

The call for a One Health approach to public safety comes at a critical time when policing, particularly with canines, is under immense scrutiny. In 2023, California state legislators proposed bill AB 742 to ban police canines for apprehension purposes, citing racially mediated usage and deadly consequences for bite victims [24]. In response, law enforcement cited claims of canine effectiveness in protecting officers and defending against lethal weapon use [25]. While the bill was moved to an inactive status due to a lack of evident support, the concerns remain apparent. This argument is substantiated by the lack of robust empirical evidence on the impacts of police canines, highlighting the need for increased institutional awareness and transdisciplinary approaches to understand and refine their societal contributions. This review will cover the landscape of police canines in the United States by addressing working dog well-being, officer safety and canine partnership, and the duo’s efforts in built environments and local neighborhoods. The discussion of known impacts as well as gaps in knowledge includes suggested approaches to integrate One Health into public safety.

## 3. Ensuring the Well-Being of Working Dogs

Many studies on working dog well-being focus on the procurement, genetic selection, training and behavioral assessments, and sustained performance of working dogs [26,27,28]. Much of this can be adapted for law enforcement needs. However, literature on animal-assisted intervention (AAI) can also be applied to police canines. AAI is a broad term to describe the utilization of an animal in a manner that is beneficial to humans [12,29]. Classically, this definition applies to assisted therapy, education, and facility activities that render services to a population. Notably, service and emotional support dogs do not fit this definition because they are procured for individual benefit.

### 3.1. Adoption of Animal-Assisted Intervention Frameworks

AAI frameworks are useful for evaluating police canines under the lens of public health. AAI must meet specific criteria, including upholding basic training standards, adhering to best practices, and regularly documenting services rendered. Such recommendations are well outlined for police K9s [30,31], though specific standards for training and (re)-certification requirements vary by state and municipality at this time [32]. With AAIs, the animal acts as an intervening force to enhance treatment or ameliorative processes from a well-trained individual; and as with therapeutic AAI, police canines themselves are the safety intervention [33]. Patrol canines have superior olfactory capabilities that serve as the primary tool for detection and tracking [34,35,36,37], and their presence is defended by law enforcement as an additional method of de-escalation against the use of lethal force [38]. Comfort dogs, akin to facility dogs, are tasked with providing aid and comfort to people, groups, and communities during times of crisis [39]. AAI services are delivered alongside their human providers (responsible person or RP) and remain within the scope of the latter’s profession. Most importantly, AAI must be goal-oriented and add value to the health and well-being of both animals and humans with minimal suffering.

There is a dearth of published data on active police dogs in the United States. As previously discussed, total numbers are unknown, let alone the diversity in working purpose, training, housing, and primary care needs. As these animals are not retained primarily for companionship, there is a deviation in the classic expectation of the social contract between man and dog. But these animals are more than just augments of human labor, and we cannot discredit the need to assure canine health and welfare. Veterinarians are uniquely qualified to aid in the scientific evaluation and documentation of the health benefits or risks of AAI [29], and this should be considered for police canines. Veterinarians should be well-positioned to comment on preventive care, training, behavioral assessments, nutritional needs, and ongoing maintenance to ensure appropriate and adequate performance [6,8]. These canine care metrics should reflect how the dog is being used by law enforcement, whether it is for comfort, scent detection, or apprehension. Other animal health and behavior expert opinions from working dog trainers and breeders can be considered. A One Health approach fostering collaboration amongst law enforcement and the veterinary community can make strides towards ensuring working dog well-being.

### 3.2. Welfare and Perceptions of Police Canines

In addition to better understanding the breadth of police canine usage in the United States, further studies are needed to determine what breed and personality characteristics are desirable for specific tasks, terrains, and localities. Additional understanding is also needed on how working dogs and handlers are optimally matched and developed to ensure appropriate care and welfare. While concerns for exploitation of police dogs are less likely [15], the benefit of collaboration between veterinarians and law enforcement is to ensure working dog welfare. A study of canine welfare perceptions in Australia reveals that police dogs have increased welfare compared to other people’s pet dogs but less than service dogs or one’s own pet dog [40]. These perceptions are often influenced by trust in associations or media representation [40], which is often widely discrepant and can fail to account for inherent breed characteristics or working purpose. A 2010 study of police canine perceptions on a Florida college campus revealed 67% to 70% of students believed the campus dogs reduced crime and deterred drug use [41]. Their social construct is shaped by the academic environment, where most individuals have neutral or no contact with the dogs. In contrast, another study found that generic images of a police officer with a dog were perceived more negatively than when the officer was pictured alone [42]. This leads to the discussion of the necessary other half of a police canine team and the canine’s primary advocate—the handler.

## 4. Promoting Officer Safety and Canine Partnership

In his publication, Chapman outlined five main advantages of police canines in North America: psychological intimidation, crime deterrence, night detection, officer safety, and public relations [3]. Determination of these advantages today is difficult to objectify given the breadth of claims. Quantitative measures of psychological intimidation and de-escalation are exceedingly challenging to capture [43], and records rely heavily on the wisdom retained by law enforcement officers [44]. At best, we turn to officer safety and public relations, where the human–animal bond framework can offer further insight.

### 4.1. The Foundation for Teamwork

The adoption of AAI literature cannot be complete without acknowledgement of the human–animal bond, namely the role of the handler in police canine units. The position of canine handler is often regarded as a status symbol, as is common with law enforcement specialty units. The criteria for becoming a police canine handler vary considerably by jurisdiction, accounting for the local culture of law enforcement [37]. Understanding handler demographics in concert with canine demographics may provide insight into appropriate and successful partnerships and the advantage of officer safety. There is no dearth of literature on human–animal interactions, but in the context of law enforcement, much less is described. Ethnographic research reveals the complexity of relationships a handler can have with a police canine, ranging from emotional—canines-as-children or canines-as-pets—to instrumental—canines-as-partners or canines-as-tools [45,46,47]. Even within their utility, there is this dichotomy in the perception of the dog as a use of force weapon or a public relations asset [42,44]. Yet these animals are more than just living instruments, with handlers describing a “bond that is unreal” [48] (pg. 363) due to the inseparable nature of their livelihoods [49].

What this reveals is that the police canine–handler bond is more than just an idiosyncratic interaction—the AVMA defines the bond as a mutually beneficial and dynamic relationship between people and animals that is influenced by behaviors that are essential to the health and well-being of both [50]. This includes, but is not limited to, emotional, psychological, and physical interactions of people, animals, and the environment [50]. The premise of AAIs is predicated on the strength of this bond [33], and there is evidence to suggest a robust bond impacts the success and well-being of a working partnership [51]. A One Health approach to capturing this dynamic spectrum and reciprocal influence may provide some new insights on police canines.

Despite recent findings rejecting a significant association between canine patrol programs and officer physical safety using theoretical modeling [44], further studies are necessary to capture the implications of not just physical but emotional and psychological safety from the human–animal bond. Law enforcement is prone to stressful and traumatic situations in the line of duty, resulting in increased vulnerability for psychological and mental health issues compared to the general population [52,53]. While qualitative data suggest temporary stress-relief benefits using therapy dogs [54], further inquiry is needed into how the human–animal bond impacts physiological measures in handlers and their working dogs, ideally with parallel study designs [55]. The data from 9/11 search-and-rescue (SAR) dogs suggest that the intimate partnership between a handler and a working dog is subject to parallel emotional dynamics [56], and this is reinforced in studies of canine–handler teams [46,47,48,49,57]. However, the intrinsically perceived value of these dogs has not been translated to community impact and measurable outcomes for public safety. One study showed that both handler and occupational stresses do influence the canine’s ability to work and that a strong emphasis on consistent training is necessary not only to improve skills but to build confidence and trust in the relationship [58]. However, more research is necessary to understand the depth of the canine–handler relationship and capacity for teamwork to inform physical and psychological safety as they approach their communities.

### 4.2. Teamwork for the Community

Laying the foundation for the canine–handler bond is essential for community work. However, evaluating the success of a handler–canine team in the community is not straightforward; for example, if an article search for a weapon results in no find, is the work a failure despite the dog doing the job it was tasked with alongside its handler? In other words, does a negative outcome always warrant a negative assessment of the team? In another example, a negative search for an explosive device is incredibly valuable, and informing the rest of the team that an area is clear of articles and/or suspects has intrinsic value for safety and efficiency of resource deployment. Other metrics for teamwork success in the community should be considered, including consistency of presence, response time, number and types of deployments^1^, and abidance to department policy, training, and certification standards [31]. These upstream metrics in preparation for canine unit implementation are equally, if not more, important to assess. An intrinsic evaluation of police canines’ training, certification, deployments, and documentation is essential to better understand how these dogs are intended to be used in the name of public safety [30]. Training provides an opportunity for law enforcement and civil society partnerships to affect police organizational culture and enhances police legitimacy. Whether it is the duration of foundational training for new recruits or the documented number of hours of in-service/maintenance training for established teams, these efforts arguably translate to successful deployment outcomes, predicated on the strength of the bond and trust in the relationship. This information is also critical for officer credibility in court. As previously mentioned, successful AAIs are goal-oriented and structured [28]; translating to law enforcement means adequate internal policy and documentation should monitor canine use outcomes and ensure best practices for minimal harm, all while promoting the human–animal bond for working success.

Simultaneously, extrinsic evaluations of the way police canines are received in a community are necessary to ensure the services being rendered are appropriate and adequate. This aligns with Chapman’s final claim of the advantage of a police canine for public relations [3]. The perceptions of police dogs depend highly on context, notably in the environments and communities in which they work in. Previously cited research supported relatively negative perceptions of the canine–handler team compared to the handler alone [42]. However, a neutral online sampling platform lacks the subjective context of communities and social environments where municipal law enforcement fits in. It also lacks the dynamic visibility of the canine–handler bond. Further research is needed to determine the extent of cultural and regional differences in interactions with law enforcement and how those attitudes extend towards working dogs in local communities. In fact, it is the canine–handler presence situated in the local community that justifies the final arm of One Health.

## 5. Community Policing Together in Built Environments and Neighborhoods

### 5.1. Law Enforcement and Public Health

There is a growing body of literature to support the intersectionality of law enforcement and public health (LEPH) [14,59], proposing that policing and public health have mutually desired outcomes, and recognizing their overlap can lead to community benefits at the intersection of health and security [60,61]. Much of this discussion within law enforcement has revolved around themes such as racial and ethnic disparities, disaster preparedness and response, mental health and occupational safety of first responders, and harm reduction for illicit drug use [59], but even crime and violence prevention may be conceptualized as a public health approach. At the local level, common public safety and security concerns may include high crime and violence rates, unsafe transportation or motor vehicle accidents, drug-related crimes, weapon offenses, and natural disasters. All of these can benefit from the appropriate involvement of law enforcement, including police canines.

At their core, both public health and law enforcement assess and regulate human behaviors [14]. They engage with physical and social environments and regularly balance the recognition of individual liberties against the need for the greater good [62]. It is within the domain of veterinary public health to promote and ensure the beneficial use of animals in society, including measures for public safety using working dogs. In combining veterinary science and law enforcement with a public health perspective, we look to the built environment to determine reciprocal relationships between the canine–handler team and the locality they serve.

Within public safety, it is imperative to consider the built environment and local neighborhoods in which municipal law enforcement work, particularly to avoid the common One Health fallacy of disregarding environmental embodiment [63]. The built environment is defined as man-made spaces designed for human activity and recreation, including buildings, public infrastructure, transportation, and open space; these have long been studied for their health impacts on how populations live, work, and play [64]. Yet structural determinants of health are intertwined with social context, including social determinants of health, such as local neighborhoods [65]. As Rock et al. [66] (p. 993) describe, “public health researchers draw on many traditions to study ecological relationships but aim to promote prevention-oriented research that takes into account the political, cultural and economic principles underpinning connections between physical environments, social environments, human populations and animal populations.”

### 5.2. The Physical Environment and Occupational Health

The physical environment poses similar occupational hazards to both handler and canine; from buildings to cityscapes to open fields, there is a complex assortment of terrain and affiliated exposures that may put the handler and canine at mutual risk for musculoskeletal injuries, heat stress and/or dehydration, and fatigue, most commonly [6]. In addition, the social community perception of law enforcement cannot be disregarded. As previously discussed, police canine and handler receptions vary based on social context [41,42], and further research is needed to investigate cultural and regional differences to ensure a socially robust approach to public safety. Longitudinal occupational health studies of police canines and their handlers are prime to capture the extrinsic environmental and social impacts on the health of the canine–handler team. Health outcomes from longitudinal studies on SAR dogs deployed in the 9/11 aftermath showed no significant differences to a control population of SAR dogs [67,68]. Similar findings were reported on a 5-year cohort study of New York Police Department police dogs deployed to the World Trade Center disaster site [69]. Though these studies are rooted in exposure to a specific incident, they draw parallels to occupational health studies of human first responders. There is value in continued health surveillance of all first responders, so further research is necessary in police canine occupational health and safety. From emerging risk factors to long-term sequelae of psychological and physiological health, a better understanding of occupational and environmental stressors is an equally important public health need for the population of police canines and their handlers.

### 5.3. The Social Environment and Public Relations

In revisiting Chapman’s claims on the advantage of having police canines for public relations [3], there is the potential for alignment with community policing, the philosophy that promotes partnerships to address conditions that impact public safety, including crime, social disorder, and fear of crime [70]. Akin to public health, community policing emphasizes prevention through safety promotion, reduction in criminogenic exposures, and community outreach. Police canines offer law enforcement high visibility and opportunities for community collaboration, whether that be through demonstrations, adult and youth academies, involvement in local school activities, and participation in major gatherings. This visibility, based on the general population perceptions of canines, is critical for fostering healthier social environments. For example, police comfort dogs are trained to provide positive interactions, leveraging the power of the human–animal bond to foster dialogue between police and the local community. These interactions can reduce anxiety in children and adults and potentially increase receptiveness to help [39]. Given the importance of this during times of trauma or tragedy, these canines have a pivotal role in community policing or crisis response teams. The importance of these non-punitive interactions cannot be underscored; a study from New England researchers determined that even one positive non-punitive contact with a uniformed officer significantly improved an individual’s perception of police legitimacy [71]. Most importantly, the study was conducted in one geographic area in concert with the presiding municipal police department [71]. A similar study design with police canines and their handlers would be meaningful in local context.

The presidential task force on policing recommends that law enforcement maintain stringent policies, training, and data collection on the use of force, and police canine units are no exception to this suggestion [72]. The recommendations for community policing applicable to police canines include transparency and accountability through deployment data, training protocols and standards, and certifications [30]. Further research is needed to understand how canine units are designed, trained, and maintained to reflect the community’s physical and social needs.

This leads to the final recommendation for policy oversight with a One Health approach. There is considerable variability in state and regional legislation on police canine training, certification requirements, and even use-of-force documentation [32]. These would benefit from implementation science and policy assessment frameworks to ensure the recommendations are effective public safety interventions. Police canine handlers have cultivated a vast body of experiential knowledge that is rarely acknowledged in the scientific literature. The translation of this knowledge to effective evidence-based practices requires an interdisciplinary approach. A summary of areas of assessment is outlined in Table 1, recognizing that methodologies should be used in more than one area. Incorporating social sciences, including participatory methodology, academia–industry partnerships, and multispecies ethnographies, assures that we grow past the biomedical roots of One Health but continue to acknowledge the human–animal–environment interface thoughtfully [73].

## 6. In Response to Iatrogenesis

It would be ignorant to not consider iatrogenesis or harms that come from police canine use of force [61]. Police have long been advocates for the patrol canine’s role in crime deterrence and de-escalation from lethal use of force [38], but strategies to characterize this benefit by measuring de-escalation remain limited [43]. While case law has deemed police canines a non-lethal use of force [4], there is acknowledgement of severe injury outcomes even when canine apprehension (i.e., bite) is used relatively infrequently [74,75].

The ratio of bites to total apprehensions, also known as a bite ratio, is an important estimate for canine use of force [76]. The published ratios range from 14% to 44%, with non-white suspects being less likely to be bitten than white suspects [77,78]. This contrasts with reports of racialized violence of police canine use-of-force [79,80]. While considerable case law has shaped policy around reasonable canine use [81], there is a noticeable dearth of available data on police canine use in this country, let alone canine apprehensions and injury outcomes in more recent years [74,82].

In recognition of this concern for iatrogenesis, the author defends that a One Health approach will be necessary to determine where and when police canines are effectively deployed in a local capacity. Public health principles of risk management and harm reduction should be incorporated to reduce the amount and impact perceived by society [13,83]. Epidemiological measures for adverse health outcomes of local violence and crime must be balanced against the adverse outcome of disease or injury from the use of these dogs. Veterinary public health has classically only considered the latter, whether it is of concern for dog bite injuries or transmission of zoonoses. Yet, without acknowledging the full spectrum of police canine and law enforcement capacity within a community, an emphasis on bites and zoonoses alone detracts from a holistic opportunity for public health and law enforcement to collaborate for local societal needs.

It is reasonable to expect that law enforcement experts, lawyers, and criminologists classically bear the responsibility for evaluating law enforcement, including police canines; yet the foundation for effective canine use can benefit from non-law enforcement expertise in domains like training, the human–animal bond, and animal health and welfare. From this, effective administrative oversight, including policy development and documentation, is necessary to define the unit’s role and responsibilities in their community. And the most important consideration is the handler, with often untapped wisdom and a realistic perspective of what is and what is not doable with canine deployments. Without these internal educational and administrative measures, there is arguably an imbalance in fostering police canine–handler well-being and their understanding of their community’s needs for public safety where the team would be most effective.

## 7. Conclusions

In their capacity to protect and serve, municipal law enforcement assures their community’s public health by virtue of addressing public safety. The decision to use police canines as a law enforcement tool can, therefore, impact community health and safety, and thus, comprehensive use is essential to define first. The manner in which police canines are procured, trained, and maintained for detection and protection can benefit from a One Health approach, including the literature from animal-assisted interventions. The human–canine bond is arguably the most mutually profound of all interspecies relationships, but this ideal social contract is marred by inconsistent perceptions of what these canines do. Though these animals are not kept for companionship, there is depth and complexity in the handler–canine relationship, ranging from pet to partner to tool, that warrants further exploration of the human–animal bond to support successful teamwork in the community. And the paucity of evidence-based research on police canines in local communities, in both experimental and observational studies, suggests that further work may excel with a new perspective. Supporting the needs and responsibilities of working dogs in society falls under the domain of veterinary public health, while advancing effective policing in society is shaped by law enforcement and public health (LEPH) agendas; together, they serve a united public health framework such as One Health. One Health implementation is championed when there is recognition that the sum is greater than any individual part; there is arguably added value to the successful use of police canines if canine well-being, officer safety and partnership, and local community needs are accounted for in a multidisciplinary fashion. Dogs have always served as man’s best friend—is there a chance for them to do more for society in the name of public safety?

## Figures and Tables

**Table 1:** Areas of assessment and possible methodologies for a One Health approach to public safety.

	Areas of Assessment	Methodologies
**Working canine**	working dog care and well-being human–animal bond	multispecies ethnography participatory methodology
**Law Enforcement officer**	policy and documentation occupational health	implementation science injury epidemiology academia–industry partnerships
**Local community** ***built environment*** ***social environment***	public perceptions public relations	risk assessment and management harm reduction phenomenological research

## Data Availability

This research does not contain new data.
